# Connexin-based signaling and drug-induced hepatotoxicity

**DOI:** 10.18053/jctres.03.2017S1.004

**Published:** 2017-02-12

**Authors:** Michaël Maes, Mathieu Vinken

**Affiliations:** Department of In Vitro Toxicology and Dermato-Cosmetology, Faculty of Medicine and Pharmacy, Vrije Universiteit Brussel, Brussels, Belgium

**Keywords:** gap junction, hemichannel, connexin, hepatotoxicity, drug-induced liver injury

## Abstract

Being critical mediators of liver homeostasis, connexins and their channels are frequently involved in liver toxicity. In the current paper, specific attention is paid to actions of hepatotoxic drugs on these communicative structures. In a first part, an overview is provided on the structural, regulatory and functional properties of connexin-based channels in the liver. In the second part, documented effects of acetaminophen, hypolipidemic drugs, phenobarbital and methapyriline on connexin signaling are discussed. Furthermore, the relevance of this subject for the fields of clinical and in vitro toxicology is demonstrated.

**Relevance for patients:** The role of connexin signaling in drug-induced hepatotoxicity may be of high clinical relevance, as it offers perspectives for the therapeutic treatment of such insults by interfering with connexin channel opening.

## 1. Introduction

**Figure 1:**
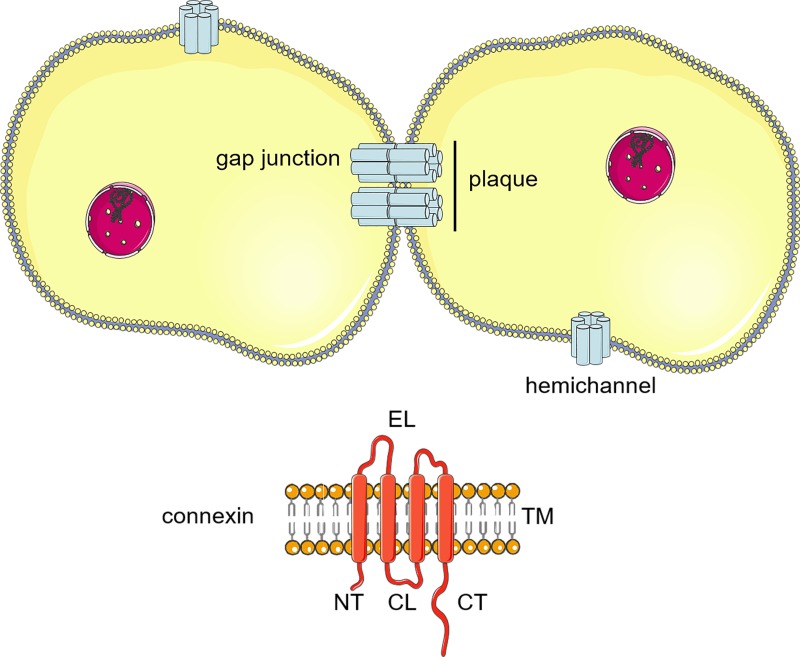
Structure of connexins and their channels. Gap junctions group in so-called plaques at the plasma membrane surface and are formed by the docking of 2 hemichannels from neighboring cells, which in turn are built up by 6 connexins. Connexins share a similar structure consisting of 4 transmembrane domains (TM), 2 extracellular loops (EL), 1 cytosolic loop (CL), 1 cytosolic carboxyterminal tail (CT) and 1 cytosolic aminotail (NT).

Gap junctions are goalkeepers of intercellular communication by mediating the passive diffusion of small and hydrophilic molecules, such as glutathione, adenosine triphosphate, cyclic adenosine monophosphate, inositol triphosphate, and ions, including calcium, sodium and potassium [1,2]. A plethora of physiological processes are regulated by substances that are intercellularly exchanged via gap junctions and hence gap junctional intercellular communication (GJIC) is considered as a key mechanism in the control of tissue homeostasis [3-13]. The liver was among the first organs in which gap junctions have been characterized [14,15]. More than 40 years ago, Goodenough isolated 2 gap junction proteins from mouse liver and called them connexins (Cx) [16]. At present, 21 different connexins have been identified in humans and rodents, all that are expressed in a cell type-specific way and named based on their molecular weight [17]. Nonetheless, they all share a common structure consisting of 4 transmembrane domains, 2 extracellular loops, 1 cytosolic loop, 1 cytosolic carboxyterminal tail and 1 cytosolic aminotail. Following synthesis, 6 connexins form a hemichannel at the plasma membrane surface, which then docks with another hemichannel from a neighboring cell to generate a gap junction [18-20] (Figure 1). This occurs at the extracellular domains, where conserved cysteine residues create disulfide bonds [21]. In recent years, it has become clear that undocked hemichannels may also provide a pathway for cellular signaling on their own independently of their role as structural precursors of gap junctions. Unlike their full channel counterparts, hemichannel communication occurs between an individual cell and its extracellular environment, yet the messengers that permeate hemichannels are very similar to those involved in GJIC [22-26]. Despite some structural variation between connexins, the first extra-cellular loop, the first transmembrane domain, the cytosolic aminotail and/or the cytosolic loop are considered to contribute to hemichannel pore opening [27]. Inherent to their participation in the maintenance of tissue homeostasis, connexins and their channels, in casu in liver, are also often involved in pathological processes, such as in liver disease and hepatotoxicity [28,29]. The present paper specifically focuses on the role of connexin signaling in drug-induced liver injury (DILI).

## 2. Connexin-based channels in liver

### 2.1 Structural properties

Cx32 is the predominant connexin in liver and is expressed by hepatocytes and sinusoidal endothelial cells next to small quantities of Cx26, which is equally produced by stellate cells and Kupffer cells [30-32]. In addition, Cx43 is present in Kupffer cells, stellate cells, sinusoidal endothelial cells, cells of Glisson’s capsule and cholangiocytes [32-37], while Cx40 and Cx37 have been detected in liver vascular cells (Table 1) [38-40]. Nevertheless, functional gap junctions have thus far only been demonstrated in hepatocytes and stellate cells [32]. In fact, gap junctions in the pericentral and periportal acinar regions typically are Cx32 homotypic and Cx32-Cx26 heterotypic channels, respectively [35,41]. This complies with the observation that Cx26 is mainly expressed in the periportal area, whilst Cx32 is evenly distributed in liver tissue [42,43].

### 2.2 Regulatory properties

Connexin signaling can be regulated by a plethora of mechanisms at the transcriptional, posttranscriptional, translational and posttranslational level. As such, 2 major kinetic sources of regulation have been described, namely short-term control (*i.e.* millisecond to minute range) and long-term control (*i.e.* hour range). They cooperate to fine-tune the degree of intercellular communication by controlling the number of channels, their functional state and their unitary permeability [44,45].

**Table 1:** Expression of connexins in liver

Connexin	Cell type	References
Cx26	hepatocytes	[164-166]
	stellate cells	[32]
	sinusoidal endothelial cells	[32]
	Kupffer cells	[32]
Cx32	hepatocytes	[32,167]
	biliary endothelial cells	[36]
	sinusoidal endothelial cells	[32]
Cx37	hepatic artery endothelial cells	[38-40]
	portal vein endothelial cells	[38-40]
Cx40	hepatic artery endothelial cells	[38-40]
	portal vein eindothelial cells	[38-40]
Cx43	biliary epithelial cells	[36, 168]
	Kupffer cells	[32-34,159]
	stellate cells	[32, 38]
	sinusoidal enothelial cells	[32, 38]
	hepatic artery endothelial cells	[38-40]
	portal vein endothelials cells	[38-40]

Long-term control of GJIC involves regulation at the transcriptional level of connexin expression [44,46]. Connexin gene promoters show binding affinity for several ubiquitous transcription factors, such as activator protein 1. Furthermore, a number of cell type-specific transcription factors govern connexin gene transcription, including hepatocyte nuclear factor 1α that regulates Cx32 production in liver [47-49]. In addition, epigenetic mechanisms, in particular histone acetylation and DNA methylation, influence connexin gene expression [46,50], as shown in liver cells [51-53].

Short-term control of GJIC, so-called gating, is regulated by a variety of factors [54-57], among which posttranslational modifications, such as *S*-nitrosylation, sumoylation and phosphorylation, are prominent ones [58,59]. *S*-nitrosylation occurs at intracellular cysteine residues and is mediated by nitric oxide, which might be the underlying mechanism of increased hemichannel opening induced by metabolic inhibition and inflammatory conditions [60,61]. Irreversible conjugation of small ubiquitin-like modifiers to lysine residues, so-called sumoylation, regulates Cx43 levels and the number of Cx43-based gap junctions at the plasma membrane [62]. Phosphorylation encompasses the addition of phosphate groups to polar amino acid side chains, among which serine, threonine and tyrosine residues. This posttranslational modification almost uniquely takes place at the cytoplasmic carboxyterminal tail. With the exception of Cx26, all known connexins are phosphoproteins that are targeted by a broad panel of kinases. The regulation of gap junction opening by phosphorylation is complex and depends on the nature of the kinase and the identity of the connexin family member [55,59,63]. Cx43 may occur as a nonphosphorylated isoform and 2 phosphorylated isoforms [64-66]. In liver, Cx43 is mostly presented as the nonphosphorylated variant in quiescent conditions [52,67].

### 2.3. Functional properties

The establishment of GJIC is indispensable for the performance of many liver-specific functions, including albumin secretion [68], glycogenolysis [69-71], ammonia removal [68], bile secretion [72,73] and xenobiotic biotransformation [74-76]. Both the constitutive and drug-induced production of cytochrome P450 isoenzymes, in particular cytochrome P450 2B6 and 3A4, require the presence of Cx32-based gap junctions [77]. Induction of cytochrome P450 1A1/2 and 2B1/2 coincides with downregulation of pericentral Cx32 protein amounts in rat [74-76]. These concomitant changes may reflect a defense mechanism to restrict the intercellular diffusion of reactive intermediates produced through xenobiotic biotransformation [74]. Gap junctions composed of Cx32 also propagate glycogenolytic responses from the periportal to the peri-central pole, in particular by controlling the intercellular trafficking of inositol triphosphate [70]. The latter activates calcium release from endoplasmic reticulum stores, in turn evoking calcium waves throughout the acinar tract [78]. Likewise, bile secretion from cholangiocytes depends on the spread of calcium waves through Cx43-based gap junctions [36,73].

Upon partial hepatectomy, gap junction coupling intensifies in the G1 phase of the cell cycle, followed by a dramatic decrease during initiation of DNA synthesis. This is paralleled by similar changes in Cx32 expression [79-89]. In the regenerating liver of rats treated with an inhibitor of mitogen-activated protein kinase, the disappearance of Cx32 is inhibited without affecting hepatocyte proliferative activity [82], which suggests that downregulation of GJIC may occur independently of cell growth. However, in the regenerating liver of Cx32^-/-^ mice, proliferative activity of the hepatocytes is not enhanced, yet the extent of synchronous initiation and termination of DNA synthesis is decreased. This may point to a supporting role for gap junctions in liver cell cycling [86,90]. The involvement of connexin signaling in liver cell growth may actually be more critical as anticipated. Thus, overexpression of Cx32 and Cx26 in rat liver epithelial cells and human hepatoma cells triggers the production of the cell cycle inhibitor p27 and the adherens junction protein E-cadherin, respectively, which, in turn, suppress proliferation [91].

Connexins and their channels have been reported to participate in different cell death processes in liver, including apoptosis [67,92,93], necrosis [94] and autophagy [95]. Interestingly, accumulating evidence suggests that connexin hemi-channels, rather than gap junctions, are involved in liver cell death. Following induction of apoptosis in primary hepatocytes, GJIC rapidly deteriorates, which is accompanied by a decay of the gap junctional Cx32 protein pool. Concomitantly, Cx32 is de novo synthesized and gathers in a hemichannel configuration. This becomes particularly evident towards the final stages of the cell death process, where Cx32 hemichannels facilitate the apoptosis-to-necrosis transition [92,96]. Along the same line, Cx43 signaling, also partly relying on hemichannels, was found to facilitate the onset of spontaneous apoptosis in cultures of primary hepatocytes [67].

## 3 Connexin-based channels and drug-induced liver injury

### 3.1. Acetaminophen

DILI is the leading cause of acute liver failure in Western countries with the vast majority being caused by overdosing with acetaminophen (APAP), a readily available analgesic and antipyretic drug [97,98]. After APAP intoxication in rodents, a switch in mRNA and protein production from Cx32 and Cx26 to Cx43 is observed [93,99]. The upregulation of Cx43 quantities is due to recruitment of Cx43-expressing inflammatory cells, but also originates from de novo production of hepatocytes [99]. In this regard, a recent study revealed that Cx43^+/-^ mice display increased liver cell death, inflammation and oxidative stress in comparison with wild type (WT) littermates after APAP overdose [99]. These results suggest that newly synthesized hepatic Cx43 may protect against APAP-induced liver toxicity. A limited number of reports have described a role for Cx32-based gap junction in APAP-triggered hepatotoxicity using genetically modified animals, albeit with contradicting outcomes [93,100-102]. In this respect, Naiki-Ito and colleagues administered APAP to Cx32-dominant negative transgenic rats and noticed decreased aminotransferase serum levels and attenuated liver damage in comparison with WT animals [93]. Likewise, ceramide synthase 2-null mice, in which Cx32 is located in the cytosol of hepatocytes and that display aberrant GJIC, are less susceptible to APAP-induced hepatotoxicity [102]. In addition, an in vitro study showed protection against synchronized necrotic cell death of attached hepatocytes originating from Cx32^+/+^ mice compared to WT hepatocytes treated with APAP. This synchronization of cell death was mediated by gap junctions formed of Cx26 and Cx32. Furthermore, APAP-sensitive male hepatocytes were protected by attachment to APAP-insensitive female hepatocytes, with this protection being dependent on gap junctions. This points to a role for gap junction-based signaling in hepatocyte death by distribution of either death signaling molecules or survival messengers between hepatocytes [94]. In contrast, another report described increased serum amino-transferase levels and more pronounced liver insults in Cx32^-/-^ mice after administration of APAP, indicating a cytoprotective function for hepatic Cx32 in APAP-induced injury, possibly linked to the trafficking of glutathione between hepatocytes *via* gap junctions [100]. This can be reconciled with the documented suppression of Cx32 production and simultaneous reduced channel activity upon exposure of hepatocytes to liver toxicants both in vitro and in vivo [29,101]. However, our group recently found that Cx32^-/-^ mice form less protein adducts 6 hours after APAP administration, which could indicate a lower metabolic activity upon genetic ablation of Cx32 [101]. Indeed, at the more upstream mechanistic platform of APAP toxicity, cell death results from protein adduct formation involving *N*-acetyl-*p*-benzoquinone imine, the toxic metabolite of APAP [103,104]. This could question the suitability of genetically deficient rodents for investigating the role of Cx32 in APAP-induced hepatotoxicity. A possible alternative is the use of inhibitors of Cx32-based gap junctions. In this regard, a small molecule inhibitor of Cx32-based gap junctions, called 2-aminoethoxy-dipenyl-borate (2-APB), was reported to protect against liver failure and death in WT mice when co-administered with APAP [105]. However, a follow-up study demonstrated that the protection was only minor or completely lost when 2-APB was administered 1.5 hours or 4-6 hours, respectively, after APAP. In addition, part of the protection was due to the solvent dimethyl sulfoxide. Furthermore, in vitro experiments showed that the protection of 2-APB was caused by inhibition of metabolic activation of APAP as well as by inhibition of the *c*-jun-*N*-terminal kinase signaling pathway and not by blocking Cx32-based gap junctions [106]. In essence, de novo produced Cx43 after APAP-induced liver toxicity seems to have a protective role, while contradictory results were found with respect to the role of Cx32-based signaling.

### 3.2. Hypolipidemic drugs

Peroxisome proliferator-activated receptor α agonists, such as clofibrate [107], nafenopin [108] and Wy-14,643 [109] are lipid-lowering agents, which drive the expression of genes involved in fatty acid transport, binding and β-oxidation in favor of proliferative activity. Chronic treatment of rodents with peroxisome proliferators has been associated with hepatocarcinogenesis due to an induction of cell proliferation coupled to a suppression of hepatocyte apoptosis [107-109]. Both in vitro [110-112] and in vivo [113,114], it has been found that clofibrate, nafenopin and Wy-14,643 reduce hepatocellular GJIC. Inhibition of GJIC by Wy-14,643 occurs in a species-specific way, since it takes place in primary cultured hepatocytes from rat, mouse and hamster, but not from monkey and human [112]. Similarly, treatment of primary hepatocytes from rat, but not from guinea pig, with nafenopin causes reversible disappearance of GJIC [110]. The latter did not result from altered Cx26 and Cx32 protein levels or modifications in the cellular localization of Cx32, but was linked to protein kinase C-mediated phosphorylation of Cx32 [111]. By contrast, clofibrate [113,115] and Wy-14,643 [114] suppressed hepatic Cx26 and Cx32 protein levels. In addition, clofibrate enhanced the appearance of Cx43 in the cytoplasm of hepatocytes [113]. Overall, peroxisome proliferators seem to perturb GJIC and alter hepatic connexin expression. Stimulation of hepatocyte proliferation by these agents has also been shown to be mediated, at least in part, by tumor necrosis factor α (TNFα) [116,117]. Therefore, a conceivable explanation is that the downregulation of the connexin signaling is driven by TNFα released in response to peroxisome proliferators [114,118,119]. Indeed, TNFα treatment has been shown to modulate GJIC and to downregulate connexin gene expression [120]. Hence, GJIC inhibition by TNFα and subsequent promotion of hepatocyte proliferation might be a possible mechanistic interpretation of the effects of peroxisome proliferators in liver.

### 3.3. Phenobarbital

Phenobarbital or phenobarbitone (PB) is an anti-epileptic drug that has sedative and hypnotic properties. It is frequently used as a model tumor promoter in rodent liver and alters the expression of a broad set of genes [116,117], of which, those related to cytochrome P450-dependent xenobiotic biotransformation have gained most attention [118]. The presence of functional gap junctions consisting of Cx32, but not of Cx26, is a prerequisite for the promotional activity of PB, since Cx32^-/-^ mice [121,122], unlike Cx26^-/-^ mice [124], are resistant to promotion of hepatocarcinogenesis by this barbiturate. Further-more, a subset of genes is differentially affected by PB in the liver of Cx32^-/-^ mice compared to their WT counterparts [123]. Interestingly, connexins are required for PB-mediated tumor promotion. It has been shown by several groups that gap junction activity becomes reduced upon administration of PB to rodents [74,113,125-128]. This is associated with abnormal frequency and size of gap junctions on the hepatocyte plasma membrane surface [129], decreased Cx32 immunoreactivity [74,125,130] and aberrant Cx32 localization [113,126], whereas Cx26 expression is not affected [74,125,126]. Both unchanged [74,128] and decreased [131,132] hepatic Cx32 mRNA levels are seen in PB-treated rodents. As shown in rodent models in vivo [128] and in vitro [133,134], the reduction of GJIC by PB occurs in a strain-specific way. Furthermore, the inhibitory effect of PB on GJIC between primary cultured mouse hepatocytes depends on xenobiotic biotransformation capacity, as it is abolished by a cytochrome P450 inhibitor [135].

### 3.4. Methapyrilene

Methapyrilene is an antihistamine with strong sedative properties that has been mainly prescribed to treat insomnia. It has been banned in most countries because of its potential to cause serious liver damage [136]. In recent years, methapyrilene has been tested in several toxicogenomics studies [136-140] and even in integrated systems toxicological trials [141] as a typical nongenotoxic hepatocarcinogen, whereby it became clear this drug induces numerous alterations in critical metabolic and signaling pathways. With respect to intercellular communication mediated by gap junctions, it has been found that the number and size of Cx32-containing gap junction plaques in liver are negatively affected upon treatment of male rats with a carcinogenic dose of methapyrilene. However, this dose also increased the occurrence of apoptosis, which may also contribute to the negative affect of methapyrilene on liver gap junctions [142].

## 4 Conclusions and perspectives

Because of its unique function and localization in the body, the liver is a primary target of toxicity induced by xenobiotics, including pharmaceuticals. Connexins and their channels are frequently involved in DILI, yet their exact role still is a matter of debate. In this light, Cx32^-/-^ mice display lack of promotion of hepatocarcinogenesis by PB [121-123] and Wy-14,643 [143], suggesting that Cx32 signaling aggravates the adverse outcome. However, most evidence points to a rather defensive function for connexin signaling [90,144-149]. Thus, a high incidence of chemical-induced liver tumors was observed in mice deficient for Cx32 [90,144] and APAP-related liver injury is increased in Cx43^+/-^ mice [99]. This discrepancy may be due, at least in part, to opposite actions of gap junctions and hemichannels. Indeed, while gap junctions are mainly associated with physiological functions, hemichannels are closed most of the time and seem to preferably open in pathological conditions [2,23,150,151]. Such differential effects of channels consisting of the same connexin building blocks are controversial and deserve further scrutiny. To add another layer of complexity, a novel class of connexin-like proteins has been identified in the last decade, namely the pannexins, which gather in a configuration identical to connexin hemichannels and that also provide an additional pathway for communication between the cytosol of individual cells and their extracellular environment [152,153]. Pannexins have been detected in a number of liver cells, in particular hepatocytes [154-159], and have been linked to lipoapoptosis [158]. Hence, pannexin signaling may also be potentially involved in drug-induced hepatotoxicity, a hypothesis that should be verified in the upcoming years.

The role of connexin signaling in DILI may be of high clinical relevance, as it offers perspectives for the therapeutic treatment of such insults by interfering with connexin channel opening. While doing is, care should be taken to develop specific channel modifiers. Besides the clinical toxicological importance, connexins and their channels are equally of interest to in vitro toxicologists. Specifically, inhibition of GJIC may represent a biomarker for the detection of nongenotoxic hepatocarcinogens, as shown for several drugs [114,142,160-163]. This could allow developing an in vitro assay for the testing of nongenotoxic carcinogenicity that might be used during early drug development [28].

## ABBREVIATIONS

2-APB: 2-aminoethoxy-dipenyl-borate
APAP: acetaminophen; Cx, connexin
DILI: drug-induced liver injury
GJIC: gap junctional intercellular communication
PB: phenobarbital
TNFα: tumor necrosis factor α
WT: wild type.
